# Specific memory B cell response in humans upon infection with highly pathogenic H7N7 avian influenza virus

**DOI:** 10.1038/s41598-020-60048-9

**Published:** 2020-02-21

**Authors:** Brenda Westerhuis, Hinke ten Hulscher, Ronald Jacobi, Josine van Beek, Marion Koopmans, Guus Rimmelzwaan, Adam Meijer, Rob van Binnendijk

**Affiliations:** 1 Centre for Infectious Disease Control (Cib), National Institute for Public Health and the Environment (RIVM), Bilthoven, The Netherlands; 2 Department of Viroscience, Erasmus MC, Rotterdam, The Netherlands; 3 Research Center for Emerging Infections and Zoonoses, University of Veterinary Medicine (TiHo), Hanover, Germany

**Keywords:** Influenza virus, Virology

## Abstract

H7 avian influenza viruses represent a major public health concern, and worldwide outbreaks raise the risk of a potential pandemic. Understanding the memory B cell response to avian (H7) influenza virus infection in humans could provide insights in the potential key to human infection risks. We investigated an epizootic of the highly pathogenic A(H7N7) in the Netherlands, which in 2003 led to infection of 89 persons and one fatal case. Subtype-specificity of antibodies were determined for confirmed H7N7 infected individuals (cases) (n = 19), contacts of these cases (n = 21) and a comparison group controls (n = 16), by microarray, using recombinant hemagglutinin (HA)1 proteins. The frequency and specificity of memory B cells was determined by detecting subtype-specific antibodies in the culture supernatants from *in vitro* stimulated oligoclonal B cell cultures, from peripheral blood of cases and controls. All cases (100%) had high antibody titers specific for A(H7N7)2003 (GMT > 100), whereas H7-HA1 antigen binding was detected in 29% of contacts and 31% of controls, suggesting that some of the H7 reactivity stems from cross reactive antibodies. To unravel homotypic and heterotypic responses, the frequency and specificity of memory B cells were determined in 2 cases. Ten of 123 HA1 reactive clones isolated from the cases bound to only H7- HA1, whereas 5 bound both H7 and other HA1 antigens. We recovered at least four different epitopal reactivities, though none of the H7 reactive antibodies were able to neutralize H7 infections *in vitro*. Our study serologically confirms the infection with H7 avian influenza viruses, and shows that H7 infection triggers a mixture of strain -specific and cross-reactive antibodies.

## Introduction

H7 avian influenza viruses represent a major public health concern, as these antigenically divergent viruses cause yearly outbreaks in poultry worldwide. Most human infections with avian influenza H7 viruses occurred after exposure to poultry, without evidence of sustained human transmission^1–8^. During previous occurrences of H7 influenza virus outbreaks among poultry, the numbers of human cases were limited and disease was mild, but severe human disease with a case fatality rate of up to 40% has been observed during the recent outbreaks of avian H7N9 in China (WHO)^9,10^. Therefore, the widespread circulation of H7N9 viruses among birds, and the potentially severe clinical impact of these zoonotic infections has led to their ranking as serious pandemic threat^11^.

Serological proof of human infection with avian influenza viruses is challenging. Several studies showed that the classical “gold standard” hemagglutination inhibition assay (HAI), used in sero-epidemiological studies, is relatively insensitive for antibodies against avian influenza viruses^12–15^, resulting in different interpretations of the HAI. Consensus in methods used in epidemiological studies is lacking, resulting in high variations in percentages in seropositives, which causes a major bias in epidemiological studies to discriminate infected cases from non-cases. During an epizootic of A(H7N7) in the Netherlands (2003) seropositivity of 85% of the persons with a virologically confirmed influenza A/H7N7 infection was found, using HAI cut off to values ≥10^2^, using a higher and more accepted cut-off of 40, only a seropositivity of 6% was found. During an outbreak in British Colombia in 2004, no H7 specific antibodies could be detected in infected persons by a similarly modified HAI assay, nor by micro-neutralization or Western blot assays (Skowronski *et al*.^3^). While within a group of patients with laboratory confirmed A(H7N9) influenza virus infection 73% seropositive patients were found using a using HAI cut off to values ≥40^16^. Similar variability on the seropositivity rates were found among poultry workers that were exposed to avian influenza viruses in other studies, ranging from 0.4% to 56% among poultry workers^17–20^. Recently, we developed protein array (PA) based serological assays to profile antibody responses to a range of influenza virus HA1 antigens, in an attempt to obtain a more granular analysis of the antibody responses in individuals exposed to human and animal influenza viruses^21–23^. While this showed the ability to measure subtype specific antibodies, the diverse reactivity of antibodies in serum samples from adults with a history of multiple influenza virus infections makes it challenging to conclude on exposure history from serum profiling. Upon vaccination with H7N9 vaccine, the induction of specific and cross- reactive H7 antibodies have been previously demonstrated^24^. To provide more evidence for the induction of specific antibody responses upon natural infection to avian influenza virus antibodies, we established an *in vitro* analysis method to gain insight into the specificity of antibodies produced from individual memory B cells^25^. The method is based on clonal dissection of the memory B cell repertoire, based on the knowledge that approximately 0.5–2% of the memory B cells are influenza virus specific^26,27^. Analysis of these antibodies from B cell clones using protein microarray gave us the opportunity to screen the available repertoire of memory B-cells in peripheral blood for a broad range of HA1 antigens to identify their strain-specificity. This will contribute to better understanding of the memory B cell response to avian (H7) influenza virus infection in humans, and provides new insights in the potential key to human infection risks. Here we used microarray to study specific antibody responses for H7 infected individuals from the highly epizootic of the highly pathogenic A(H7N7), which led to infection of 89 persons and one deadly case^1,2^. We investigated subtype- specificity of antibody responses in serum for H7 infected individuals, and subsequently selected from these individuals a number to determine the presence and specificity of H7 specific memory B cells in peripheral blood during convalescence.

## Results

### Protein array HA1 serum titers in H7N7 infected individuals

**Figure 1:**
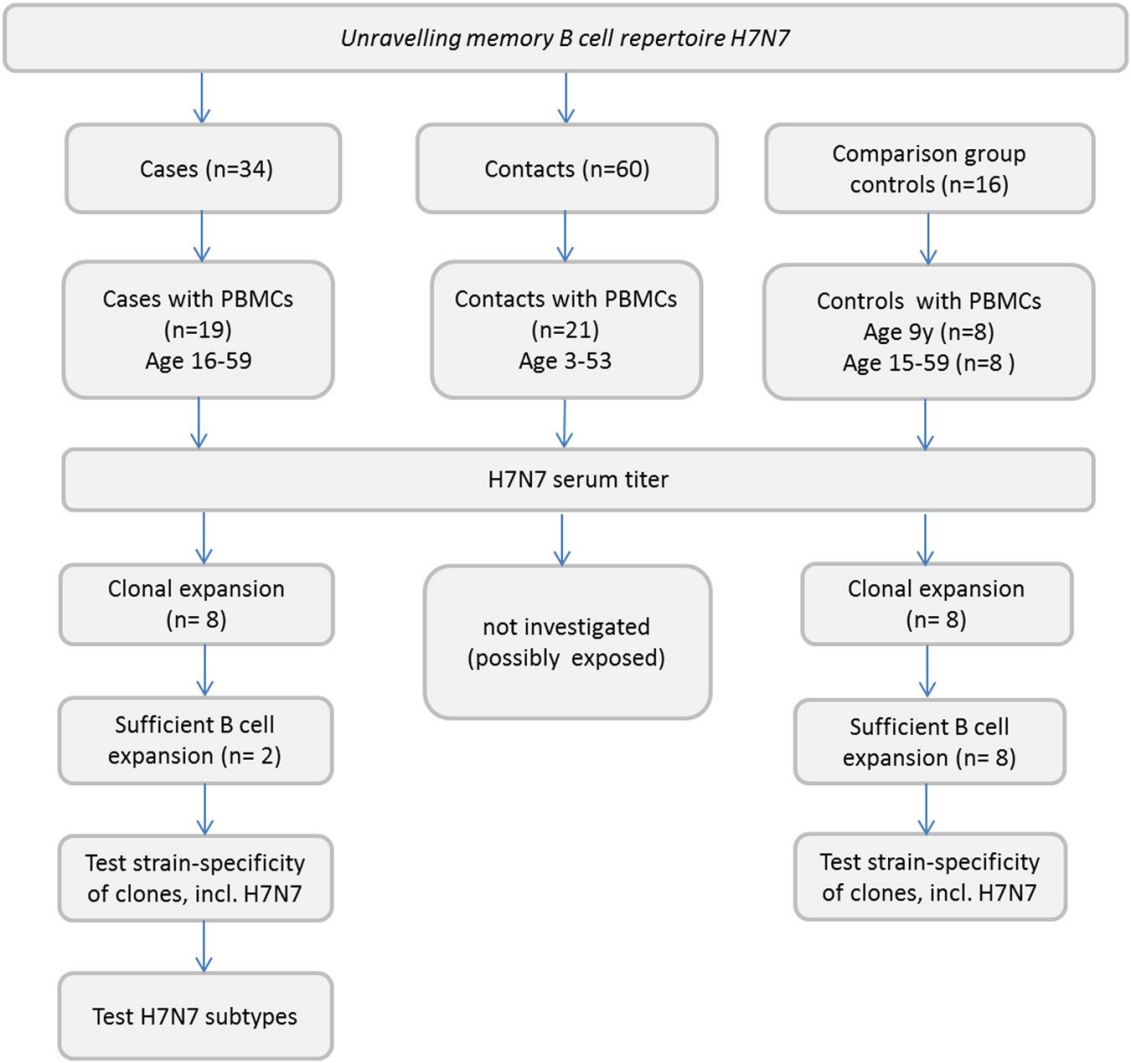
An outline of the study groups and selections.

**Figure 2:**
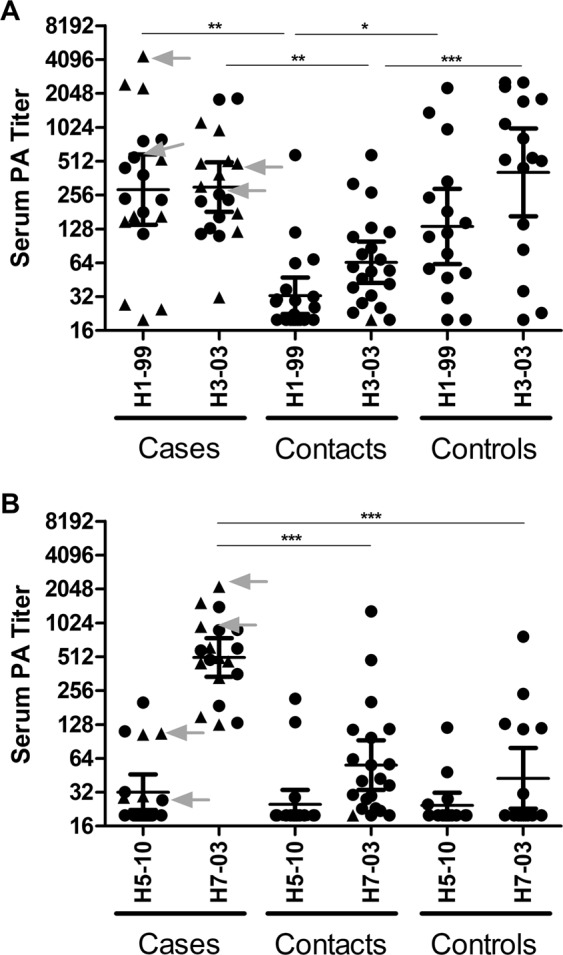
Protein microarray (PA) serum titers. (**A**) Serum profiles of H7N7 cases with confirmed infection. Two cases used for B cell analyses are depicted in grey. (**B**) Serum profiles of contacts of the H7N7 infected cases. Individuals with confirmed additional vaccination upon the outbreak are indicated with a triangle, individuals with no information on additional vaccination are indicated with a circle (**C**). Serum titers of individuals upon A(H1N1)pdm09 pandemic outbreak individuals with confirmed A(H1N1)pdm09 infection are indicated with a triangle, individuals with no confirmed infection are indicted with a circle. Error bar indicates the GMT with 95% CI. P values are calculated using unpaired student t-test on log-transformed serum titers. The serum titers of the cases used for B cell cultures are indicated with arrows.

We first characterized serum profiles by PA using recombinant HA1 proteins representing human and avian influenza virus type A strains. We included the representative avian A(H7N7)2003, a second avian A(H5N1)2010 to which these individuals are not likely to be exposed, and two seasonal subtypes A(H1N1)1999 and A(H3N2)2003 representative for the two seasonal vaccination strains administered during the outbreak in 2003. Sera available from 19 infected individuals and 21 contacts from the epizootic of highly pathogenic avian A(H7N7) in the Netherlands in 2003 were tested in this PA. We supplemented these with samples collected during the A(H1N1)pdm09 outbreak as an additional comparison group (Fig. 1). We showed high titers against HA1 of A(H7N7)2003 for all cases (GMT = 503) (Fig. 2A). In the case group we also found clear high GMTs (≥80) against HA1 of the vaccine representative subtypes A(H1N1)1999 and A(H3N2)2003 (Fig. 2B), but not to another avian subtype, A(H5N1)2010 (Fig. 2A). In all contacts of the H7 infected individuals, the GMT for these representative strains were <80, including the A(H7N7)2003 (GMT = 55) (Fig. 2A). In the additional comparison group we found a GMT > 80 for A(H1N1)1999, A(H3N2)2003, but a GMT < 80 for avian A(H7N7)2003 (GMT = 42) (Fig. 2B). While the GMT’s were low, some individuals had antibodies binding to A(H7N7)2003 HA1 in the contact group (n = 6). This might indicate low level transmission of H7N7 from cases to contacts^28^, though we also found some H7 reactivity in the comparison group (n = 5, titer>80), which might indicate the presence of cross reactive antibodies. Therefore, we cultured and cloned memory B cells from stored PBMC samples of two PCR-confirmed cases. Both showed high serum protein array PA titers against H7N7 (Case 1 = 1418, Case 2 = 617), but low or absent HAI (Table 1).

**Table 1:** Case description used for further analyses.

	PCR	Age	Confirmed vaccination Upon outbreak	HAI titer H7N7	HAI titer H1N1	HAI titer H3N2
Case 1	Pos.	35 y	NA^*^	20	≥640	160
Case 2	Pos.	20 y	Y	<10	≥640	160

^*^Information from this subject not available.

### B-cell memory repertoire of cases infected with A(H7N7)2003

**Figure 3:**
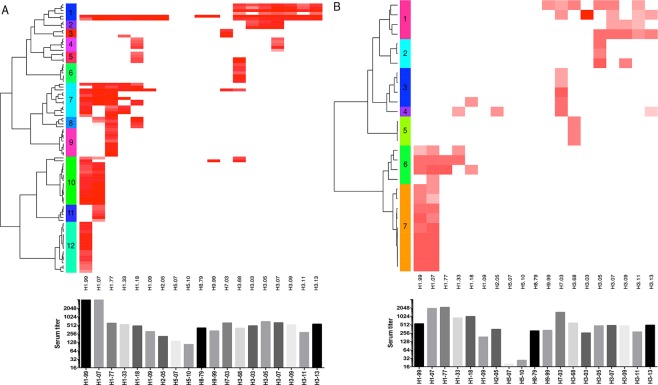
Strain-specific reactivity of clones isolated from case 1 (**A**) and case 2 (**B**), presented as heatmap (upper figure). Each line in the heatmap represents the HA1 reactivity of clones produced by a single memory B-cell precursor cell. Heatmaps are generated by log2-transformation of the RFU and by applying hierarchical clustering, clusters are generated and indicated using different colors. The bar graph (below) represents the serum titer of both cases.

We applied a clonal dissection method by seeding replicates of a fixed low number of B cells (≤500 cells per well) to dissect the frequency and specificity of influenza virus specific B cell clones from cases and from the control group, with the aim to provide evidence for the existence of specific A(H7N7) B cells in cases. In total we detected 95 influenza HA1 positive B cell cultures for case 1and 28 positive for case 2, which corresponds to a precursor frequency of 0.0412% and 0.0179% with respect to all CD19+ B cells seeded for each donor. Furthermore, we can consider positive antibody reactivity to originate from a single memory B cell clone, as the majority of B cell cultures at an input as low as 500 cells per well were antibody negative^27^. Assuming ~20–40% of the CD19+ B cells to be memory B cells and ~30–40% of these to be capable of clonal antibody production in culture^27,29^, this correlates to a memory B cell fraction that is approximately 10x higher, so 0.412% and 0.179%. These numbers are in line with published data on influenza virus memory B cells^26,27^. In our control A(H1N1)pdm09 pandemic cohort we found on average a slightly lower CD19+ B cell frequency of 0.0103% (Table 2), possibly due to a mixture of previously A(H1N1)pdm09 infected and non-infected individuals (range 0.0015–0.025%). To visualize the homology and heterology of each B cell reactivity, we organized them into a heatmap according to their strain-specific reactivities and applied a hierarchical clustering method (Canberra distance and Ward’s clustering method) to categorize them into different clusters. Each arm of the heatmap represents HA1 antibody reactivity of a particular clone. For case 1 the method generated 12 clusters from the 95 HA1 reactive clones, for case 2 we found a minimum of 7 different clusters on the basis of 28 clones. It should be noted that the reactivity profile within each cluster is still heterogenic, pointing to possibly even more differences in the epitope specificity of individual B cells than what is currently separated by the applied dendrogram cluster method at a bootstrap value of 100. In both donors we found HA1 clones exclusive to A(H7N7)2003, for case 1 we detected 4 clones (0.0017%), which corresponds to 4.2% of all HA1 reactive clones (Fig. 3A, cluster 3) and for case 2 we detected 6 clones (0.0035%, corresponding to 21.4% of all HA1-reactive clones (Fig. 3B, cluster 3). In both cases we also found clones specific for A(H7N7)2003. Such clones were not detected in any of the B cell cultures generated from the donors of the A(H1N1)pdm09 control cohort (Table 2). However, we did find some heterosubtypic A(H7N7)2003 clones (0.0008%, n = 14, 4,5% of the HA1 specific memory cells), but only in those older than 15 years of age.

**Table 2:** B-cell precursor frequencies from different groups.

	HA1 positive clones (frequency %)*	HA1 H7N7 heterosubtypic positive clones (frequency %)*	HA1 H7N7 homosubtypic positive clones (frequency %)*
H7N7 case 1	95 (0.0412)	2 (0.0009)	2 (0.0009)
H7N7 case 2	28 (0.0179)	4 (0.0023)	2 (0.0012)
H1N1 pandemic cohort(n = 8, 15–59 y)	305 (0.0103)	14 (0.0008)	0

^*^Frequency: percentage to total number of CD19+ cells.

High antibody reactivity in serum to HA1 antigens representing seasonal H1 and H3 strains as well as their potential to cross-react was confirmed at the clonal level as visualized by heatmap level. In case 1 a broad H1 response, (Fig. 3A, cluster 7, 8) including single antibody reactivities towards A(H1N1)1977 (cluster 9), A(H1N1)1999 (cluster 12), and A(H1N1)2007 (cluster 11) were found. Clones reacting against A(H1N1)1999 and A(H1N1)2007 were represented in cluster 10 (Fig. 3A). Surprisingly we also detected a large number of clones reactive with A(H1N1)1918 (Fig. 3A, cluster, 4, 5, 7 and 8), an influenza strain to which none of the individuals was exposed earlier in life. Two clusters of broadly cross reactive H1 clones are shown of which three clones also showed high reactivity with A(H1N1)pdm09 (Fig. 3A, cluster 1 and 7). Clones within cluster 1 showed reactivity with both H1 and H3 strains, one of which also showed cross reactivity with H8 and H9, but not with any of H7 or H5 (Fig. 3A). In case 2 (Fig. 3B), most clones mainly showed reactivity with A(H1N1)1999 and A(H1N1)2007 (Fig. 3B cluster 7) of which some also showed reactivity with A(H1N1)1977 and A(H1N1)1933 (Fig. 3B, cluster 6). As shown in Case 1 some low cross-reactivity with A(H1N1)1918 is shown (Fig. 3B cluster 3 and 6). Notable, both single reactivities were found against A(H3N2)1968 (Fig. 3B, cluster 5) and A(H3N2)2005 (Fig. 3B, cluster 2) which are both types to which this 20-year old donor has not been exposed.

### Cross reactivity of A(H7N7)2003 HA1 reactive clones

**Figure 4:**
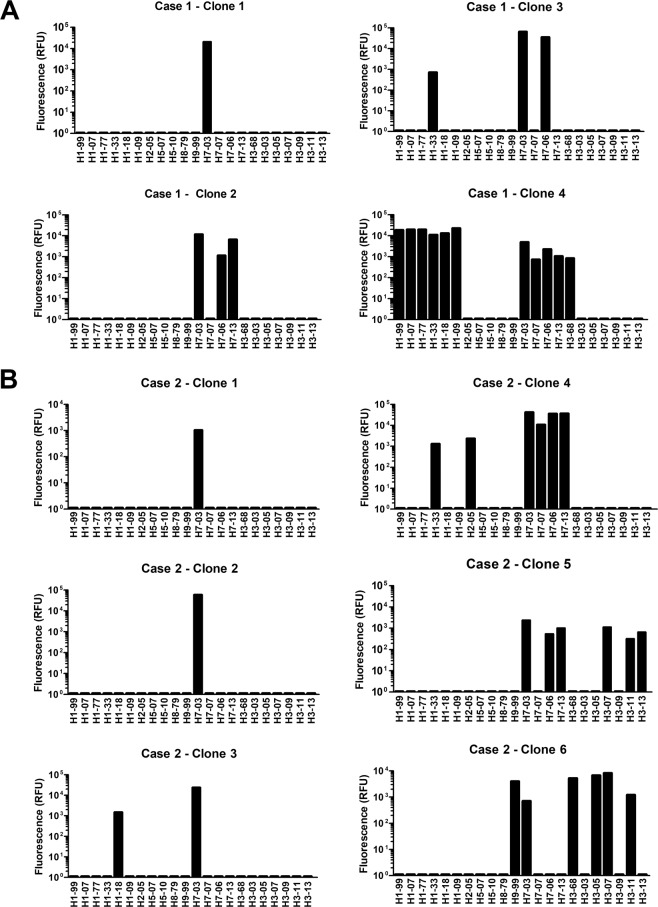
Cross reactivity of the A/H7N7/2003 reactive clones isolated with different H7 strains, measured with the protein microarray. Each graph represents an individual A(H7N7) reactive clone.

**Figure 5:**
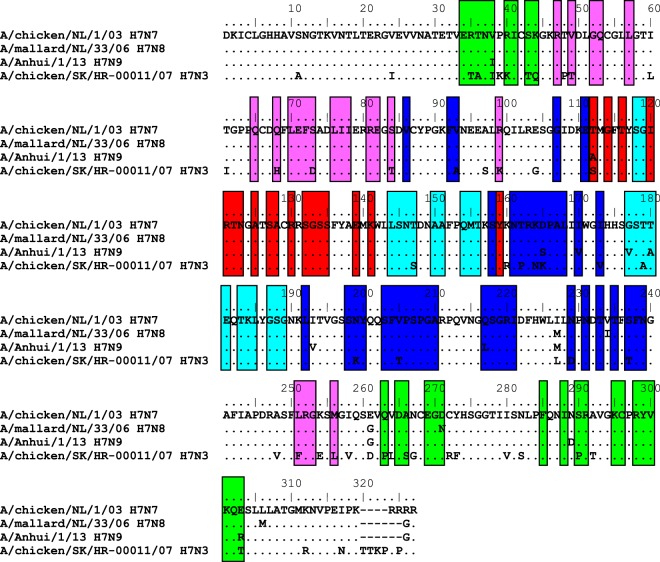
Amino acid alignment of HA1 of the H7 strains included in this study. Alignment and plotting for the figure were performed in BioEdit software version 7.2.5. Antigenic epitope sites have been taken from Liu et al. 2015. Amino acid positions included in antigenic epitope A in red, B in light blue, C in green, D in dark blue and E in purple.

Anti-H7 2003 positive B cell cultures from the cases were subsequently tested against HA1 from different H7 strains, including H7N8 A/mallard/NL/2006, H7N3 A/chicken/SK/2007, and H7N9 A/Anhui/2013. The full (cross-) reactive profile of each of the A(H7N7)2003 reactive clones is depicted in Fig. 4. In total, three out of ten clones showed strain-specific reactivity for A(H7N7)2003 case 1 clone #1 and case 2 clones #1 and #2. Two clones positive for A(H7N7)2003 from case 1 (Ab 4) and from case 2 (Ab 4) showed cross-reactivity against all the other H7 HA1 proteins tested. Three of the clones showed cross reactivity with A(H7N8)2006 and/or A(H7N9)2013, case 1 (Ab2 & 3) and case 2 (Ab 5). Amino acid sequence and antigenic epitope^22^ analyses revealed that the HA1 sequences of more cross-reactive H7N8 A/mallard/NL/2006 and H7N9 A/Anhui/2013 HA1 showed higher similarity with A(H7N7)2003 HA1 than H7N3 A/chicken/SK/2007 HA1 (Fig. 5). The H7N8 A/mallard/NL/2006 showed only a single aa change, and the H7N9 A/Anhui/2013 showed single aa differences in 4 antigenic epitopes of H7N9 compared to A(H7N7)2003. This while the H7N3 A/chicken/SK/2007 showed in all 5 antigenic epitopes up to 9 amino acid differences compared to A(H7N7)2003. Notable is that both clones cross-reactive with A(H7N3)2007 also showed cross reactivity with H1N1 strains, case 1 (Ab 4) and case 2 (Ab 3 & 4), though some cross reactivity was shown by two other A(H7N7)2003 as well. Clones cross-reactive with other group 2 HA1 antigens were also shown, one Ab showed cross reactivity with A(H7N8)2006 and A(H7N9)2013, and reactivity with particular H3 strains (Ab 5). A second Ab also showing cross reactivity with H3 strains, showed only reactivity with the A(H7N7)2003 subtype, also the only Ab being cross reactive with another class avian HA1 protein A(H9N2)1999.

### Neutralization capacity of the A(H7N7)2003 HA1 reactive clones

We tested three A(H7N7)2003 positive and one influenza negative B cell culture for neutralization of A/Netherlands/33/2003 H7N7. As the human serum samples were not able to neutralize the H7N7 virus^2^ we included sera from ferrets vaccinated with A(H7N9)2013 as a positive control^30^. None of the clones tested were able to neutralize A(H7N7) viral infection *in vitro* in a standard micro-neutralization test, while the ferret sera were able to neutralize the infection efficiently. Furthermore, similar B cell culture supernatants generated from patients with positive laboratory confirmation of A(H1N1)pdm09 infection and tested at similar dilutions were capable to neutralize A(H1N1)pdm09 virus (data not shown).

## Discussion

Multiplex serology provides new opportunities to investigate strain-specific and cross-reactive seroconversion upon influenza virus infection. Previous studies directed at A(H1N1)pdm2009 specific seroconversion showed a strong correlation between microarray titers and standardized HI titers^21,25^. This correlation is less present in the case of human infection with avian A(H7N7) influenza virus, and thus may not be valid for each strain, probably due to underperformance of the existing serology assays. Here, we showed high titers (>80) in all the cases with PCR and culture confirmed A(H7N7) infection compared to 85% with the improved HAI, but only when the HAI cut off titer was reduced to 10^2^. Antibody binding to the HA1 as measured with the PA does not necessarily inhibit hemagglutination or lead to virus neutralization. Further studies are needed to confirm these findings.

By oligoclonal culture of viable B cells we isolated H7-2003-HA1 reactive clones from two cases with confirmed A(H7N7) infection. In the context of all B cell reactivities against influenza virus, we found a relative low frequency of A(H7N7) memory precursor cells upon infection. This could be related to lower precursor frequencies of memory B cells that we recovered from peripheral blood during early convalescence, or indicate a relative poor immunogenic response to the avian H7 strain. In this study the cases suffered from conjunctivitis, which might indicate a local infection not inducing a strong B-cell memory response. Immunization with H7 was previously shown to induce specific B-cell responses^31^, though most H7 vaccine candidates showed poor immunogenicity in humans without adjuvant or a prime boosting regime, as judged by HAI and micro-neutralization^32–39^. In contrast, A(H1N1)pdm09 seasonal vaccines are highly immunogenic in adult subjects without adjuvant. However, naive children need also prime boosting regime for seasonal vaccination.

We further tested the H7 strain-specificity of the supernatants harvested from the clones against several other H7 strains, including the recent A(H7N9)2013 subtype. We recovered at least four different H7 epitopal reactivities from the H7-specific memory B cells that were isolated from these cases, i.e. clones that were positive for A(H7N7)2003 only, clones which cross-reacted with H7N8, and clones showing cross reactivity with both H7N8 and A(H7N9)2013, some of which also showed low reactivity with A(H7N3)2007. The latter could be explained by the differences in antigenic epitopes shown compared to the other H7 HA1 proteins that were included in this study. One supernatant positive for A(H7N7)2003 Ab from case 1 showed high reactivity against A(H1N1) antigens, which might indicate broadly cross reactive antibodies including H7 strains, not induced by an H7 strain. In addition, we found several other non H7 clones which are cross reactive covering group 1 and 2 antigens (Table 3), including two antibodies covering all H1 and all H3 antigens present, which can be explained by common epitopes. A possible explanation is that the cross-reactive clones are induced by boosting of H1 and H3 memory B-cells due to A(H7N7) infection driven by previous exposures to different H1 and H3 strains, also referred to as “original antigenic sin”. Upon vaccination with A(H7N9) the group of Thornburg *et al*. showed indeed the induction of H7 specific monoclonal antibodies (mAbs) containing low number of somatic hyper mutations, whereas they also isolated less-specific H7 reactive mAbs possessing a higher degree of somatic hyper mutations suggesting that arose from memory B cells induced by prior influenza virus infection. Here we acknowledge similar reactive binding patterns, though the non-clonal background of our B cell cultures does not allow a further sequence analysis of the heavy and light chains of the generated strain-specific mAb involved.

**Table 3:** Influenza virus antigens. Pandemic strains indicated in bold. Accession numbers as provided by the manufacturer.

	Subtype	Strain	Designation	
Group 1	H1N1	A/New Caledonia/20/99	H1–99	AJ344014
	H1N1	A/Brisbane/59/2007	H1-07	KP458398
	H1N1	A/USSR/92/1977	H1-77	CY009284
	H1N1	A/WS/1933	H1-33	VU08904
	H1N1	A/South Carolina/1/18	H1-18	AF117241
	H1N1	A/California/6/2009	H1-09	KC781723
	H2N2	A/Canada/720/05	H2-05	DQ009917
	H5N1	A/Egypt/N03072/2010	H5-10	CY062484
	H5N1	A/Cambodia/R0405050/2007	H5-07	AEN68936
	H8N4	A/pintail duck/Alberta/114/1979	H8-79	ABB87729
	H9N2	A/Guinea fowl/Hong Kong/WF10/99	H9-99	AY206676
Group 2	H7N3	A/chicken/SK/HR-00011/2007	H7-07	ACA25329
	H7N7	A/Chicken/Netherlands/1/03	H7-03	AAR02639
	H7N8	A/mallard/NL/33/2006	H7-06	EPI182113
	H7N9	A/Anhui/1/2013	H7-13	EPI439507
	H3N2	A/Aichi/2/68	H3-68	V01085.1
	H3N2	A/Wyoming/3/03	H3-03	AY531033
	H3N2	A/Wisconsin/67/X-161/2005	H3-05	ACF54576
	H3N2	A/Brisbane/10/2007	H3-07	KM978061
	H3N2	A/Perth/16/2009	H3-09	GQ293081
	H3N2	A/Victoria/361/2011	H3-11	KM821347
	H3N2	A/Switserland/97/2013	H3-13	*

*Only sequence provided by Immune Technology Corp. (immun-tech.com).

In the contact group we found 6 donors with serum antibodies against A(H7N7)2003, three of these were positive in the HAI, which might indicate that exposure to H7N7 confirmed infected persons in the house-hold have led to (asymptomatic) transmission of H7N7 virus^28^. In contrast, our additional control group has most likely not been in contact with avian influenza virus, though we detected heterosubtypic A(H7N7)2003 serum antibodies in two adult individuals, indicating cross-reactivity induced by previous exposures to human influenza viruses, notably A(H1N1)pdm2009. Also, additional seasonal vaccination during the A(H7N7)2003 outbreak may have increased such cross-reactive responses during convalescence in cases. The serological profiles on the microarray indeed suggests that most cases have received vaccination, showing high serum titers against group 1 and group 2 HA1, with all GMT ≥ 100 except for A(H1N1)pdm2009, and A(H5N1). Our data on B-cell clones suggests the same, as the frequency of clones in both cases were dominated by the presence of clones specific for A/New Caledonia/20/99 and the closest related subtype A/Brisbane/59/2007 represented on the array.

The isolated H7 clones were not able to neutralize infection *in vitro*, which is in line with the lack of virus neutralization of the sera isolated from these cases. In literature, several studies showed limited neutralization by sera from H7 infected individuals^2,40^. This not necessarily means that the antibodies are non-functional, as non-neutralizing functions of antibodies have been implicated in immune protection. For example the interaction between antibodies on the surface of influenza virus infected cells and the Fcɣ-receptor(R) on natural killer (NK) cells results in killing of those cells, a process called antibody-dependent cellular cytotoxicity (ADCC)^41–43^. These functions needs to be further investigated for avian influenza viruses. Vaccination studies showed that indeed both neutralizing and non-neutralizing antibodies could provide protection in mice^31,44^. Finally, the concentration of H7 specific antibodies derived from the B cells may have been too low to reach efficient neutralization. Although, we have demonstrated neutralization against human H1N1 and H3N2 infection *in vitro* with antibodies obtained from similar experiments (data not shown).

In summary, in this study we isolated specific clones upon natural infection in humans with A(H7N7) avian influenza viruses, providing a means for retrospective detection of infection following known exposure with avian influenza virus. These clones showed diverse cross-reactivity, including the recent endemic H7N9, which is a major burden in Asian countries. Despite that we were not able to devote a function yet to these clones, we provided an alternative to isolate and characterize these rare A(H7N7) clones.

## Methods

### Study population

We selected 19 sera collected from laboratory confirmed A(H7N7) infected persons during the epizootic in the Netherlands, of which PBMCs were available (group 1, age range 16–59), and 21 sera (group 2, age range 3–53) from contacts of these persons^1,2^. Blood specimens of group 1 and 2 were taken approximately one month after onset of illness of cases in group 1. Nine out of nineteen cases had confirmed receiving seasonal influenza virus vaccination during the outbreak, as described in Meijer *et al*.^2^ An A(H1N1)pdm09 pandemic cohort group was added as a third and separate comparison group to this study, which consisted of 8 children (10 years old) and 8 older individuals (15–59 years old), for which serum and PBMC had been obtained within a year after the start of the A(H1N1)pdm09 pandemic. We specifically mixed the comparison group with sera from children that were obtained from a longitudinal Bordetella Pertussis study (Trial ISRCTN64117538)^45^, to meet the broad age distribution of the contact group. The adult serum samples of the comparison group were extracted from a 2009 pandemic outbreak study^22^. An outline of the study groups and selections are shown in Fig. 1. From the cases and from the comparison group we selected 8 donors each for clonal expansion. We selected 8 individuals from the adult A(H1N1)pdm09 cohort as these best represented the case group as these were more likely never exposed to avian influenza virus compared to the contact group.

The Dutch Medical Ethics Committee approved the studies and each person participating in the study gave informed consent, if subjects were under the age of 18 a parent and/or legal guardian provided additional informed consent. All experiment and methods were performed in accordance with relevant guidelines and regulations at the Institute of Public Health (RIVM), in the Netherlands.

### Protein microarray

Recombinant HA1 proteins of 19 different influenza virus type A strains (Table 3), expressed in mammalian cell lines (ImmunTech, SinoBiologicals) (Table 3), were printed onto 64-pad nitro-cellulose slides (ONCYTE AVID, Grace Bio-Labs, Bend, Oregon, USA) by a Arrayjet Marathon printer (Arrayjet Ltd, Roslin, UK)). Strain selection was based on the availability of recombinant HA1 antigens, and to span major strains from group 1 and 2 influenza viruses. Incubation and washing was performed as previously described^22^. Serum samples were tested with four-fold serial dilutions, starting at 1:40 dilution in Blocking Buffer (Thermo Fisher Scientific Inc., Rockford, USA), containing 0.1% Surfactant (Thermo Fisher Scientific Inc.). B-cell culture supernatant samples were pooled 4 cultures in Blocking Buffer-0.1% Surfactant. Single supernatants from positive pools were repeated, each 10  µl in Blocking Buffer. Fluorescent slides were measures using a Tecan Powerscanner (Tecan Group Ltd, Männedorf, Switzerland). The titer for each serum sample was defined as previously described^22^. The quality and reproducibility was defined using international standard NIBSC serum 07/150 as positive control, which showed reactivity with all spotted antigens. A titer of 20 was assigned to all samples in which the first dilution (1:40) was negative. B-cell supernatants showed lower background signals on the protein array compared to sera. Based on testing antibody-negative culture supernatants, a fluorescent signal of 1000 represented baseline antibody reactions, and this value was subtracted from all fluorescent signals. Validation of the B cell supernatants of each experiment was carried out by testing B cell culture supernatants for the concentrations of IgG antibodies.

### Virus neutralization

Experiments were performed at BSL-3. B-cell supernatants that were positive for antibodies against H7-HA1 were two-fold serial diluted (from 1:4) in virus growth medium (MEM medium, Gibco) supplemented with 40 µg/ml gentamycin, 0.01 M Tricin (Sigma- Aldrich) and 2 µg/ml TPCK-treated trypsin (Sigma-Aldrich). The sample dilutions were incubated for 2  hours at 37 °C with 100 TCID^50^ of H7N7 A/Netherlands/33/03^1^. A back titration of the virus stock was prepared by a ½ log^10^ serial dilution. The samples were transferred to a 96 wells plate containing a confluent monolayer of MDCK cells. After 2  hours incubation at 37 °C the virus and control mixtures were removed and serum free medium was added. Plates were incubated for 4–5 days, until the back titration plate reached cytopathic effect (CPE) at a titer of 100 TCID50. As a control experiment we performed a neutralization experiment with homologous A(H1N1)pdm09 positive B-cell supernatants.

### B cell memory cell culture

B cells were purified from cryopreserved PBMCs, and subsequently expanded *in vitro* as essentially described^25,46^. Briefly, B-cells were isolated via CD19 positive selection (Easysep, Stemcell technologies, Cologne, Germany). Our approach is based on clonal dissection of the memory B cell repertoire as previously described (Pinna *et al*. 2009). Briefly we seeded a sufficiently low concentration of 300/500 B cells per well to guarantee clonality of the influenza virus specific memory B cell fraction, based on the assumption that approximately 20–40% of the CD19+ B cells were memory B cells and that around 0.5% of these were influenza specific^26,27,29^. At these input concentrations, the percentage of influenza virus specific antibody positive B cell cultures per donor never exceeded 37%, which confers a condition by which influenza virus-specific antibodies mostly originate from a single memory B cell precursor representing a single antigen or epitope specificity, as previously documented (27). We refer to these as clones in this manuscript. At least 576 replicates of these cultures were generated per donor to provide a sufficient number of clones for repertoire analysis. Because influenza virus antibody positive cultures harbor only one memory precursor, the precursor frequency could be calculated as the ratio of influenza virus positive cultures to the total input of CD19+ cells per donor. Memory B cells were stimulated and expanded in the presence of low concentrations (500/well) gamma irradiated CD40L expressing murine fibroblasts L cells, 3  μg/ml CpG ODN2006 (Isogen Life Sciences, Utrecht, Netherlands), 10  ng/ml interleukin 2 (IL-2) (Miltenyi Biotec, Leiden, Netherlands) and 10  ng/ml IL-10 (BD Pharmingen, San Diego, USA). After 4–5 days of incubation at 37 °C, the stimulation medium was refreshed with medium containing 10  ng/ml IL-2 and 100  ng/ml IL-21 (Miltenyi Biotec). Supernatants were harvested at day 11. Total IgG production of individual B cell cultures was checked by a standard human IgG ELISA (data not shown). Anti-influenza virus reactivity was tested by protein microarray.

### Data analysis B-cell cultures

Data of B-cell supernatants is shown as relative fluorescent signal (RFU) at a single dilution (1:4), ranging from a fixed background signal of 1000 to the maximum readout of 65535. The RFU was log2-transformed using GraphPad Prism software version 6.04, these transformed values were plotted using R version 3.2.5, in which the quantitative levels were translated to color densities. The data was visualized in a heatmap, generated by applying hierarchical clustering (Canberra distance and Ward’s clustering method) as described in Freidl *et al*.^47^.

### Statistical analysis

Data were analyzed using GraphPad Prism. An unpaired student t-test on log-transformed samples was used for statistics on GMT data^48^.
