# Publisher Correction: Specific memory B cell response in humans upon infection with highly pathogenic H7N7 avian influenza virus

**DOI:** 10.1038/s41598-020-62303-5

**Published:** 2020-03-20

**Authors:** Brenda Westerhuis, Hinke ten Hulscher, Ronald Jacobi, Josine van Beek, Marion Koopmans, Guus Rimmelzwaan, Adam Meijer, Rob van Binnendijk

**Affiliations:** 1 Centre for Infectious Disease Control (Cib), National Institute for Public Health and the Environment (RIVM), Bilthoven, The Netherlands; 2 Department of Viroscience, Erasmus MC, Rotterdam, The Netherlands; 3 Research Center for Emerging Infections and Zoonoses, University of Veterinary Medicine (TiHo), Hanover, Germany

Correction to: *Scientific Reports* 10.1038/s41598-020-60048-9, published online 21 February 2020

In the original version of this Article, the author Rob van Binnendijk was incorrectly indexed. This error has now been corrected.

